# The moderating effect of neuroticism on the relationship of migration status and health-related quality of life in Germany: a population-based study

**DOI:** 10.1186/s12955-025-02380-0

**Published:** 2025-06-06

**Authors:** Arieja Farugie, Lisa-Marie Maukel, Ana N. Tibubos

**Affiliations:** 1 Department of Diagnostics in Healthcare & eHealth, Trier University, Trier, Germany; 2 Department of Psychology, Goethe University Frankfurt, Frankfurt, Germany; 3 Department of Psychosomatic Medicine and Psychotherapy, University Medical Center Mainz, Mainz, Germany

**Keywords:** Health-related quality of life, Mental health, Physical health, Neuroticism, Migration, Refugee

## Abstract

**Purpose:**

Ample research exists on the association of migration status and health-related quality of life (HRQoL). Specific personality traits linked to the Big Five such as neuroticism, have been found to be predictive of migrants’ intercultural success. This study aimed to analyze the moderating effect of neuroticism on migration status and HRQoL in a representative German sample.

**Methods:**

Data from the German Socioeconomic Panel (GSOEP) (*N* = 29,968) were analyzed. The sample included Germans without migration background (*n* = 19,749), immigrants (*n* = 3,491), foreigners residing in Germany (*n* = 2,460), and refugees (*n* = 4,268). Gender-stratified moderation analyses were performed using General Linear Models with HRQoL (SF-12; mental and physical health) as outcome, migration status as predictor, and neuroticism (BFI-S) as moderator.

**Results:**

Refugees reported the lowest HRQoL (*F*(3, 29192) = 27.54, *p* <.001). Refugees also displayed the lowest neuroticism scores. Significant moderation effects of neuroticism on the relationship between migration status and SF-12 mental HRQoL scores were observed, especially for refugees (β = 0.05, *CI* [0.01 – 0.10], *p* =.03) and immigrants (β = 0.04, *CI* [0.00 – 0.08], *p* =.03; *R²* = 0.064). Women had lower SF-12 scores compared to men across all groups. Additionally, there was a significant three-way interaction effect for female refugees with neuroticism on mental HRQoL (β = − 0.24; *CI* [-0.34 – − 0.14]; *p* <.001).

**Conclusion:**

The results corroborated research pointing out differences in HRQoL depending on migration status and gender. Refugees seem to benefit particularly from low neuroticism, in terms of emotional stability, as a psychological resource.

**Supplementary Information:**

The online version contains supplementary material available at 10.1186/s12955-025-02380-0.

## Introduction

Migration is a leading and dynamic topic in public health and social development of societies. In recent years, there has been a substantial increase in migration to Europe, with a notable surge of 16% since 2015. In 2020, Germany, in particular, stands out as the European country with the largest foreign-born population, hosting almost 18 million migrants including refugees as of 2020 [1]. Reasons for migration are mostly driven by familiar and economic reasons, such as seeking better opportunities and a higher quality of life for themselves and their families [2].

The association between migration and health, especially mental health, and overall well-being, has been extensively investigated. Numerous studies have linked the migration process to stressful life events, identifying migration as a potential risk factor for health issues [3, 4]. In Europe, migrant populations generally exhibit poorer physical health compared to host populations. Conditions such as heart disease, allergies, and occupational illnesses are more prevalent among migrants [5].

Regarding mental health, refugees in Europe experience higher rates of psychological distress compared to non-migrants. The most common mental health conditions in refugees include post-traumatic stress disorder (~ 30%) and depression (~ 30%) [6]. Beyond traumatic experiences, the length of stay in the host country also influences mental health outcomes, suggesting that ongoing challenges such as discrimination, social isolation, and occupational downgrading exacerbate these issues [5, 7–9].

Importantly, the migration journey—particularly the exposure to trauma—and the post-migration legal and social barriers, such as healthcare access, can differ significantly between refugees and work migrants. Even when refugees are legally entitled to healthcare, they often face additional challenges, such as administrative hurdles, language barriers, and limited health literacy, which hinder their ability to utilize healthcare services [5]. These obstacles contribute to poorer health outcomes for refugees. In contrast, several studies suggest that work migrants tend to report better health and quality of life compared to non-migrants (e.g [10]). This disparity points to the presence of self-selection biases, where healthier individuals are more likely to migrate for work, and highlights the need to consider specific population characteristics—such as refugee status versus work migration—when evaluating the relationship between migration and health outcomes.

These differences in healthcare access and health outcomes between refugees and work migrants underscore the importance of examining Health-Related Quality of Life (HRQoL), a key patient reported measure that reflects not only physical and mental health but also social functioning, which collectively influences an individual’s overall well-being [11–13]. Assessing HRQoL across diverse populations is challenging due to potential differences shaped by cultural influences in perception, reporting, and experience of health. For example, a multiethnic cross-sectional cohort (*N* = 7,198) of the Singapore general population consisting of Chinese (*n* = 4,873), Malay (*n* = 1,167) and Indian (*n* = 1,158) revealed significant differences in overall HRQoL and its dimensions among these ethnic groups [14]. In addition, inequivalence of psychosocial constructs such as depression [15], anxiety, or HRQoL may arise [16], when the interpretation of the construct is shaped by cultural influences [17]. Notably, the Short Form Health Survey (SF-12) assessing the physical and mental health dimensions of HRQoL, has demonstrated measurement invariance across various demographic groups (e.g., gender, age, migration status) [16].

In Germany, the Socio-Economic Panel (GSOEP) has been used to investigate HRQoL differences among diverse migration populations. A recent study found that German immigrants and individuals without a migration background initially had comparable mental HRQoL levels. However, only immigrants experienced a decline over time, particularly younger immigrants. Second-generation immigrants exhibited higher baseline physical HRQoL, while first-generation immigrants had lower baseline levels compared to non-immigrants. All groups experienced similar decreases in physical HRQoL over time, influenced by age, with females generally having lower mental and physical HRQoL [18]. Other studies found lower mental HRQoL but higher physical HRQoL of refugees compared to a German normative sample [16]. Germans, with and without a migration background, had better mental HRQoL than refugees, while refugees, particularly men, showed better physical health. Female gender and older age predicted lower physical and mental HRQoL. Younger and male individuals generally displayed higher HRQoL in both dimensions [16]. These variations underscore the importance of considering subgroups differences, especially between voluntary and involuntary migrants [19–21].

Moreover, personality traits play a significant role in predicting and understanding individual differences in HRQoL. The five-factor model of personality (Big Five or OCEAN model) — encompassing openness, conscientiousness, extraversion, agreeableness, and neuroticism — is the most widely used model for describing personality [22]. Typically, poor well-being is associated with high neuroticism, and low levels of agreeableness, conscientiousness, and extraversion [23]. This pattern is particularly relevant after critical life events such as migration. Personality also influences social perceptions and interactions, especially during intercultural challenges. Traits linked to the Big Five, such as emotional stability (low neuroticism), open-mindedness, flexibility, social initiative, and cultural empathy, predict migrants’ intercultural success [24]. Thus, personality traits serve as sources of both vulnerability and resilience, affecting cultural adaptation and well-being among migrants [25].

Neuroticism, in particular, has been extensively studied for its moderating effect on health outcomes, self-perceived health, and mental health [26]. However, the impact of neuroticism on HRQoL is not uniform across all individuals or population subgroups. For instance, studies on migration-related factors have shown inconsistent associations between neuroticism and migration intentions [27, 28]. Few studies have explored the moderating effect of neuroticism on HRQoL among migrants so far [29–31].

The present study aims to analyze the moderating effect of neuroticism on mental and physical HRQoL among migrants (immigrants and foreigners residing in Germany) and refugees, compared to the host population, taking gender differences into account.

By unraveling the complex interplay between personality traits, particularly neuroticism, and HRQoL among migrants and refugees, this study will provide valuable insights to inform interventions and support strategies aimed at promoting the well-being and overall QoL of these populations.

Our hypotheses are as follows:

### H1

Refugees report the lowest HRQoL compared to other migration status groups.

### H2

Neuroticism is negatively associated with HRQoL in all migration groups.

### H3

Neuroticism moderates the relationship between migration status and HRQoL, with stronger effects for certain migration statuses (e.g., refugees).

### H4

Women report lower HRQoL than men across all migration groups, with female refugees reporting the lowest HRQoL.

### H5

(Exploratory): We examine whether migration status, neuroticism, and gender interact to shape HRQoL.

## Method

Data from the GSOEP [32] served as the primary basis for our detailed analysis. In particular, we utilized data from 2018 (wave bi), while information on personality facets was drawn from 2017 (wave bg). To capture migration-specific contexts, we additionally integrated data from the IAB-SOEP (waves M1 and M2 “Migration Sample”) [33] and IAB-BAMF-SOEP (waves M3 to M5 “Refugee Sample”) [34], thereby ensuring a more comprehensive examination of diverse population groups.

### Sample and measures

The study population was stratified into four groups based on their migration status: (1) *Germans* without migration background, representing the host population in Germany; (2) *immigrants*, representing the host population in Germany with migration background, (3) *foreigners*, representing migrants temporarily residing in Germany, and (4) *refugees*, representing asylum seekers and those with refugee status. The specific countries of origin for participants in each group are listed in Supplement Table S1. In addition to migrations status, sociodemographic characteristics such as gender, age, and employment were reported in sample description.

HRQoL was evaluated using the GSOEP version of SF-12 [35], which includes two correlated subscales: mental (MCS) and physical health (PCS), totaling 12 items. The SF-12 is a well-established and validated instrument, with high scores indicating better HRQoL. Its psychometric properties, including the reliability of the correlated two-factor structure or composite score, have been thoroughly evaluated [16, 35]. In this study, both the mental health (ω = 0.93) and physical health (ω = 0.91) subscales showed very high reliability. Additionally, the SF-12 has been evaluated for measurement invariance regarding migration status, country of origin, survey language, gender, and age [16]. We also tested measurement invariance across our study groups, suggesting scalar measurement invariance (see Supplement S2 and Table S2).

The GSOEP version short form of the Big Five Inventory (BFI-S) was used to assess the Big Five dimensions [36]. Derived from the Big Five Inventory [37] this short scale has been widely employed in personality studies using GSOEP data. It measures neuroticism, extraversion, openness, conscientiousness, and agreeableness through 15 items, with higher scores indicating higher levels of each personality trait. The psychometric validity of the BFI-S has been established, and its reliability is considered acceptable to good for a concise inventory [36]. In our study, reliability scores were similarly good (ω_Openesse_ = 0.70; ω_Conscientiousness_ = 0.67; ω_Extraversion_ = 0.71; ω_Agreeableness_ = 0.57; ω_Neuroticism_ = 0.62).

### Statistical analysis

Data analysis was performed with Rstudio [38] (Supplement Table S3). Descriptive statistics and Pearson’s correlations were computed to assess variable relationships, with significance tests conducted at a 5% level, two-sided. Hemphill’s guidelines were employed to interpret correlation coefficients: below 0.20 indicated weak associations, 0.20 to 0.30 indicated moderate associations, and above 0.30 indicated strong associations [39]. To evaluate influences on SF-12 and its subscales (PCS and MCS), hierarchical regression was employed, sequentially adding predictors [40]. A moderation analysis, following Hayes’ regression-based methodology [41], examined interaction effects between predictors and moderators on SF-12, PCS, and MCS [42, 43].

Initially, migration status was tested as a single predictor for SF-12, PCS, and MCS (Model 1, Hypothesis 1). In Model 2, the association between neuroticism and SF-12 was examined (Hypothesis 2), along with its moderating effect on migration status (Hypothesis 3). Gender was then included in Model 3 to assess its relationship with HRQoL (Hypothesis 4). Finally, Model 4 tested a three-way interaction among gender, neuroticism, and migration status (Hypothesis 5).

Beta estimates with confidence intervals and effect sizes (*R²* and adjusted *R²*) were reported for all models. Effect sizes were interpreted as follows: *R²* (or adjusted *R²*) of 0.02 as small, 0.13 as medium, and 0.26 as large [32, 35]. Given our sample size, effects ranging from 0.02 to 0.13 were anticipated, aligned with Aberson’s guidance for small effects in samples *N* < 1,000 with a statistical power of 0.90 [43].

## Results

### Sample characteristics

Table 1 displays sociodemographic sample characteristics. In the total sample (age *M* = 47, *SD* = 17.62) and across all migration statuses except refugees, women outnumber men. Refugees are the youngest group, followed by immigrants and foreigners. The average age gap between the oldest (Germans) and youngest (refugees) groups is 16 years. Most individuals in all groups except refugees are full-time employees (37–40%). In contrast, over 73% of refugees are unemployed.

**Table 1:** Sociodemographic characteristics of the total sample and stratified for migration status

	Total *N* = 29,968	Germans *n* = 19,749	Immigrants *n* = 3,491	Foreigners *n* = 2,460	Refugees *n* = 4,268
Gender, *n* (%)					
Female	15,541 (51.9)	10,585 (53.6)	1,934 (55.4)	1,332 (54.1)	1,690 (39.6)
Male	1,4427 (48.1)	9,164 (46.6)	1,557 (44.6)	1,128 (45.9)	2,578 (60.4)
Employment, *n (%)*					
Full-time	10,316 (34.4)	7,480 (37.9)	1,399 (40.1)	910 (37.0)	527 (12.3)
Part-time employed	4,397 (14.7)	3,214 (16.3)	556 (15.9)	377 (15.3)	250 (5.9)
Occasionally employed	1,703 (5.7)	1074 (5.4)	287 (8.2)	166 (6.7)	176 (4.1)
Partial retirement	55 (0.2)	50 (0.3)	3 (0.1)	2 (0.1)	-
Vocational training	822 (2.7)	487 (2.5)	145 (4.2)	46 (1.9)	144 (3.4)
Voluntary service	60 (0.2)	50 (0.3)	10 (0.3)	-	-
Internship	44 (0.1)	-	-	1 (0.0)	43 (1.0)
Unemployed*	12,528 (41.8)	7,358 (37.3)	1,085 (31.1)	957 (38.9)	3,128 (73.3)
NA	43 (0.1)	36 (0.2)	6 (0.2)	1 (0.0)	-
Age (in years), *M (SD)*	46.98 (17.62)	51.06 (17.95)	41.56 (15.67)	43.32 (13.58)	34.60 (10.97)
Age groups, *n* (%)					
$\:\le\:$ 25	4,061 (13.5)	2,172 (11.0)	652 (18.7)	207 (8.4)	1,030 (24.1)
26–35	4,774 (15.9)	2,132 (10.8)	680 (19.5)	550 (22.4)	1,412 (33.1)
36–45	5,658 (18.9)	2,951 (14.9)	876 (25.1)	727 (29.6)	1,104 (25.9)
46–55	6,074 (20.3)	4,410 (22.3)	586 (16.8)	536 (21.8)	542 (12.7)
56–65	4,286 (14.3)	3,480 (17.6)	404 (11.6)	260 (10.6)	142 (3.3)
$\:>$ 65	5,115 (17.1)	4,604 (23.3)	293 (8.4)	180 (7.3)	38 (0.9)

Note: *The “unemployed” category includes individuals who are not only unemployed but homemakers and retirees without gainful employment. Individuals who could not be clearly assigned to one of the migration status groups (*n* = 338) are not depicted

Table 2 presents statistics (means and standard deviations) of the Big Five subscales, SF-12, and its MCS and PCS subscales, stratified by migration status and gender. Refugees, particularly males, show lower mean scores on the neuroticism (N) scale compared to other groups. Additionally, refugees report lower average HRQoL (SF-12) scores compared to other groups. German males report lower PCS compared to other migration statuses. Overall, females consistently report significantly lower HRQoL and PCS scores. Specifically, female refugees exhibit the lowest HRQoL scores.

**Table 2:** Scale statistics with gender-specific analyses of the SF-12,* the MCS and PCS subscales*,* and the big five scales*

	SF-12^a^	MCS^b^	PCS^c^	O^d^	C^e^	E^f^	A^g^	*N* ^h^
	M (SD)							
**Total**	3.50 (0.51)	3.49 (0.43)	3.51 (0.77)	19.85 (4.28)	17.37 (2.85)	14.83 (3.43)	16.43 (2.96)	11.34 (3.73)
Female ^a – h^	3.45 (0.52)	3.46 (0.44)	3.43 (0.79)	19.94 (4.28)	17.60 (2.73)	15.08 (3.38)	16.71 (2.87)	12.08 (3.72)
Male	3.56 (0.49)	3.52 (0.43)	3.59 (0.74)	19.75 (4.28)	17.12 (2.95)	14.54 (3.47)	16.12 (3.04)	10.52 (3.56)
**Germans**	3.50 (0.49)	3.52 (0.40)	3.48 (0.77)	19.56 (4.12)	17.14 (2.81)	14.63 (3.42)	16.11 (2.86)	11.33 (3.68)
Female ^a – h^	3.45 (0.50)	3.48 (0.41)	3.42 (0.79)	19.74 (4.16)	17.46 (2.70)	14.98 (3.37)	16.50 (2.79)	12.05 (3.68)
Male	3.56 (0.47)	3.56 (0.39)	3.56 (0.74)	19.35 (4.06)	16.78 (2.88)	14.23 (3.43)	15.66 (2.87)	10.50 (3.50)
**Immigrants**	3.54 (0.51)	3.49 (0.43)	3.58 (0.76)	19.89 (4.27)	17.23 (2.93)	14.98 (3.43)	16.31 (2.96)	11.83 (3.81)
Female ^a – h^	3.48 (0.51)	3.45 (0.44)	3.51 (0.77)	20.15 (4.21)	17.51 (2.85)	15.27 (3.42)	16.62 (2.94)	12.50 (3.80)
Male	3.61 (0.49)	3.54 (0.42)	3.67 (0.73)	19.56 (4.33)	16.87 (2.99)	14.62 (3.40)	15.92 (2.94)	10.98 (3.65)
**Foreigners**	3.54 (0.54)	3.51 (0.44)	3.56 (0.79)	19.67 (4.61)	17.68 (2.73)	14.87 (3.38)	16.65 (2.86)	11.82 (3.73)
Female ^a – d; g & h^	3.48 (0.55)	3.48 (0.44)	3.49 (0.81)	19.94 (4.71	17.79 (2.62)	15.00 (3.35)	16.91 (2.85)	12.42 (3.72)
Male	3.60 (0.51)	3.55 (0.43)	3.65 (0.77)	19.33 (4.47)	17.55 (2.85)	14.71 (3.42)	16.32 (2.83)	11.06 (3.62)
**Refugees**	3.44 (0.58)	3.35 (0.54)	3.54 (0.78)	23.25 (4.17)	19.69 (2.08)	16.58 (3.20)	19.73 (1.99)	9.97 (3.79)
Female ^a; c; d; g; h^	3.35 (0.62)	3.33 (0.57)	3.37 (0.83)	22.76 (4.63)	19.80 (1.96)	16.40 (3.23)	19.87 (1.88)	10.59 (3.90)
Male	3.51 (0.54)	3.36 (0.52)	3.65 (0.73)	23.51 (3.88)	19.63 (2.14)	16.67 (3.18)	19.66 (2.04)	9.65 (3.69)

Note. SF-12 = Summary Score of SF-12; MCS = Mental Component Summary of SF-12; PCS = Physical Component Summary of SF-12;

BFI-S domains: O = Openness; C = Conscientiousness; E = Extraversion; A = Agreeableness; N = Neuroticism; ^a – h^ = significant t-test for gender

Correlation analyses of the total sample (*n* = 29,968) reveal significant negative correlations between neuroticism and SF-12 (*r(23*,*746)* = − 0.278, *p* <.001), MCS (*r(23*,*746)* = − 0.224 *p* <.001), and PCS (*r(23*,*746)* = − 0.240, *p* <.001). The other domains of the BFI-S are not correlated with SF-12, nor its subscales (see Table 3). Neuroticism is positively correlated with gender (*r(23*,*746)* = 0.207; *p* <.001), indicating higher neuroticism levels in females compared to males. Among Germans (*n* = 19,749), similar negative correlations between are found between neuroticism and SF-12 (*r(17*,*446)* = − 0.298; *p* <.001), MCS (*r(17*,*446)* = − 0.257; *p* <.001), and PCS (*r(17*,*446)* = − 0.245; *p* <.001), and a positive correlation between neuroticism and gender (*r(17*,*455)* = 0.209; *p* <.001), see supplement Table S4 (correlation analyses stratified for migration status Table S4-S7). For immigrants and foreigners, these negative correlations are weaker, especially in the MCS subscale (see supplement Table S5 & Table S6). Among refugees (*n* = 4,268) correlations between neuroticism and SF-12 (*r(1*,*513)* = − 0.228; *p* <.001), MCS (*r(1*,*513)* = − 0.137; *p* <.001), and PCS (*r(1*,*513)* = − 0.246; *p* <.001) are notably weaker compared to other groups, especially for MCS. Similarly, the correlation between neuroticism and gender is weaker among refugees (*r(1*,*513)* = 0.099; *p* <.001; see supplement Table S7).

**Table 3:** Correlation matrix of gender,* age*,* and SF-12*,* its subscales MCS and PCS*,* and the big five scales including neuroticism for the sample independent of migration status*

	Age	SF-12	MCS	PCS	O	C	E	A	*N*
**Gender**	− 0.003	− 0.110^***^	− 0.085^***^	− 0.096^***^	0.026^***^	0.090^***^	0.084^***^	0.104^***^	**0.207** ^***^
**Age**		**− 0.204** ^***^	0.107^***^	**− 0.324** ^***^	− 0.063^***^	0.088^***^	− 0.094^***^	− 0.010	− 0.035^***^
**SF-12**			**0.700** ^***^	**0.921** ^***^	0.023^***^	0.047^***^	0.061^***^	0.044^***^	**− 0.278** ^***^
**MCS**				**0.365** ^***^	− 0.061^***^	0.040^***^	0.004	0.032^***^	**− 0.224** ^***^
**PCS**					0.063^***^	0.039^***^	0.077^***^	0.039^***^	**− 0.240** ^***^
**O**						**0.220** ^***^	**0.339** ^***^	0.180^***^	− 0.101^***^
**C**							**0.234** ^***^	**0.309** ^***^	− 0.127^***^
**E**								0.102^***^	− 0.165^***^
**A**									− 0.150^***^

Note. SF-12 = Summary Score of SF-12; MCS = Mental Component Summary of SF-12; PCS = Physical Component Summary of SF-12;

BFI-S domains: O = Openness; C = Conscientiousness; E = Extraversion; A = Agreeableness; N = Neuroticism; gender was coded 1 = male, 2 = female; computed correlation used pearson-method with listwise-deletion; correlation coefficients > ± 0.150 are in bold

*** = *p* <.001; ** = *p* <.01; * = *p* <.05

In Model 1 (Table 4, Hypothesis 1), immigrants show significantly higher scores on SF-12 (β = 0.07, *p* <.001) and the PCS subscale (β = 0.13, *p* <.001) compared to Germans, while reporting significantly lower MCS values than Germans (β = − 0.06; *p* =.001). Similarly, foreigners also have significantly higher SF-12 (β = 0.07, *p* =.001) and PCS scores (β = 0.06, *p* <.001) than Germans, with no significant effect observed for MCS. In contrast, refugees consistently report significantly lower scores across all scales compared to Germans (SF 12: β = − 0.11, *p* <.001; PCS: β = − 0.07, *p* <.001; MCS: β = − 0.39, *p* <.001). The regression models indicate effect sizes ranging from *R²-*adj = 0.002 for PCS to *R²-*adj = 0.017 for MCS, with MCS having an eight times higher value than PCS, though overall effect sizes are very low (Table 4).

**Table 4:** Regression analyses of SF-12,* MCS*,* and PCS on migration status*

	SF 12		MCS		PCS	
*Predictors*	β (*CI*)	*p*	β (*CI*)	*p*	β (*CI*)	*p*
Intercept	0.00 (-0.01 – 0.02)	**< 0.001**	0.06 (0.05 – 0.08)	**< 0.001**	− 0.03 (-0.05 – − 0.02)	**< 0.001**
Immigrants	0.07 (0.04 – 0.11)	**< 0.001**	− 0.06 (-0.10 – − 0.03)	**0.001**	0.13 (0.09 – 0.16)	**< 0.001**
Foreigners	0.07 (0.03 – 0.11)	**0.001**	− 0.01 (-0.06 – 0.03)	0.505	0.10 (0.06 – 0.15)	**< 0.001**
Refugees	− 0.11 (-0.15 – − 0.08)	**< 0.001**	− 0.39 (-0.42 – − 0.35)	**< 0.001**	0.07 (0.04 – 0.10)	**< 0.001**
Observations	29,196		29,411		29,364	
*R*^*2*^ / *R*^*2*^ adjusted	0.003 / 0.003		0.017 / 0.017		0.002 / 0.002	

Note. SF-12 = Summary Score of SF-12; MCS = Mental Component Summary of SF-12; PCS = Physical Component Summary of SF-12; CI = Standardized CI; reference group = Germans

**Fig. 1:**
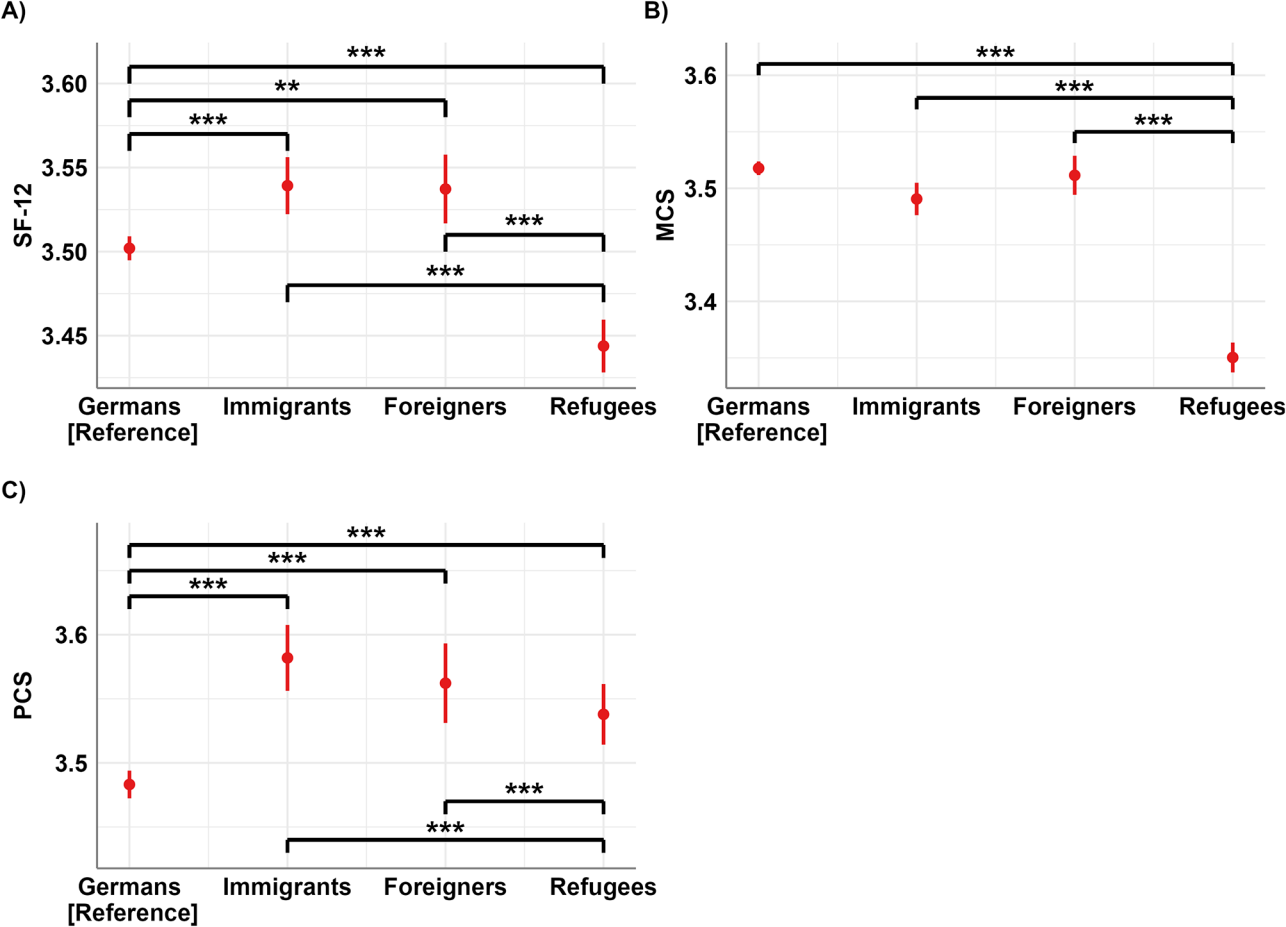
Comparative analysis of SF-12, MCS, and PCS across migration statuses. Note: Predicted values are depicted by red dots (error bars red lines), which represent the health-related quality of life (HRQoL) for the migration status: Germans (reference), immigrants, foreigners, and refugees. SF-12 panel **A**); MCS panel **B**); PCS panel **C**). The black lines indicate significant comparisons between the migration statuses, and asterisks denote the statistical significance levels from post hoc Tukey tests comparing group means, with *** indicating *p* <.001 and ** indicating *p* <.01

In Fig. 1A immigrants and foreigners show significant differences in SF-12 from refugees (both *p* <.001, but no significant difference is found between immigrants and foreigners. Germans report significantly lower SF-12 scores than immigrants and foreigners (both *p* <.001) and significantly higher scores than refugees (*p* <.001) (Hypothesis 1). Figure 1B shows no significant differences between immigrants and foreigner for MCS. Refugees report significantly lower MCS scores than the other migration statuses (*p* <.001). Germans report significantly higher MCS scores than immigrants (*p* <.01) but show no significant difference compared to foreigners. In Fig. 1C, Germans exhibit significantly lower PCS scores compared to other migrations statuses (*p* <.001), while no significant differences are observed among immigrants, foreigners, and refugees.

In Model 2 (Table 5), immigrants (β = 0.12; *p* <.001) and foreigners (β = 0.13; *p* <.001) have higher SF-12 scores compared to Germans. However, refugees demonstrate a significantly lower score on SF-12 (β = − 0.20; *p* <.001). Neuroticism also exhibits a significant negative association with SF-12 (β = − 0.29; *p* <.001) (Hypothesis 2). There is no significant interaction between the migration status and neuroticism for SF-12.

The moderation analysis of the PCS subscale revealed significant main effects of both migration status and neuroticism. Specifically, immigrants had higher PCS scores compared to Germans (β = 0.17; *p* <.001), as did foreigners (β = 0.15; *p* <.001). However, there was no significant association for refugees with PCS scores compared to Germans. Neuroticism had a negative main effect on PCS (β = − 0.25; *p* <.001). Similar to SF-12, there was no significant interaction observed between neuroticism and migration status for PCS (see Table 5).

The analysis examining the moderating effect of neuroticism on the association of migration status and the MCS subscale showed a significant main effect for refugees (β = − 0.47, *p* <.001) indicating lower MCS scores compared to Germans. Neuroticism also had a significant main effect on the MCS subscale (β = − 0.25; *p* <.001). Additionally, there was evidence of a moderating effect (β = 0.04, *p* =.030) of neuroticism among immigrants and the MCS subscale. Likewise, there was a positive significant interaction among refugees and neuroticism on MCS (β = 0.05; *p* <.001; Table 5, Hypothesis 3).

**Table 5:** Regression analyses of SF-12, MCS, and PCS on migration status with neuroticism as moderator

	SF 12		MCS			PCS	
*Predictors*	β (*CI*)	*p*	β (*CI*)	*p*	β (*CI*)		*p*
Intercept	− 0.01 (-0.03 – 0.00)	0.118	0.03 (0.02 – 0.05)	**< 0.001**	− 0.03 (-0.05 – − 0.02)		**< 0.001**
Immigrants	0.12 (0.08 – 0.16)	**< 0.001**	− 0.03 (-0.07 – 0.01)	0.089	0.17 (0.13 – 0.21)		**< 0.001**
Foreigners	0.13 (0.08 – 0.17)	**< 0.001**	0.03 (-0.01 – 0.08)	0.180	0.15 (0.11 – 0.20)		**< 0.001**
Refugees	− 0.20 (-0.26 – − 0.15)	**< 0.001**	− 0.47 (-0.52 – − 0.42)	**< 0.001**	− 0.01 (-0.06 – 0.05)		0.815
Neuroticism	− 0.29 (-0.31 – − 0.28)	**< 0.001**	− 0.25 (-0.26 – − 0.23)	**< 0.001**	− 0.25 (-0.26 – − 0.24)		**< 0.001**
Immigrants x Neuroticism	0.02 (-0.02 – 0.06)	0.257	0.04 (0.00 – 0.08)	**0.030**	0.01 (-0.03 – 0.04)		0.713
Foreigners x Neuroticism	0.02 (-0.03 – 0.06)	0.407	0.04 (-0.01 – 0.08)	0.098	0.01 (-0.04 – 0.05)		0.775
Refugees x Neuroticism	0.03 (-0.02 – 0.08)	0.261	0.05 (0.01 – 0.10)	**0.030**	0.01 (-0.04 – 0.06)		0.612
Observations	24,032		24,189			24,137	
*R*^*2*^ / *R*^*2*^ adjusted	0.084 / 0.084		0.065 / 0.064			0.064 / 0.063	

Note. SF-12 = Summary Score of SF-12; MCS = Mental Component Summary of SF-12; PCS = Physical Component Summary of SF-12; CI = Standardized CI; reference group = Germans

**Fig. 2:**
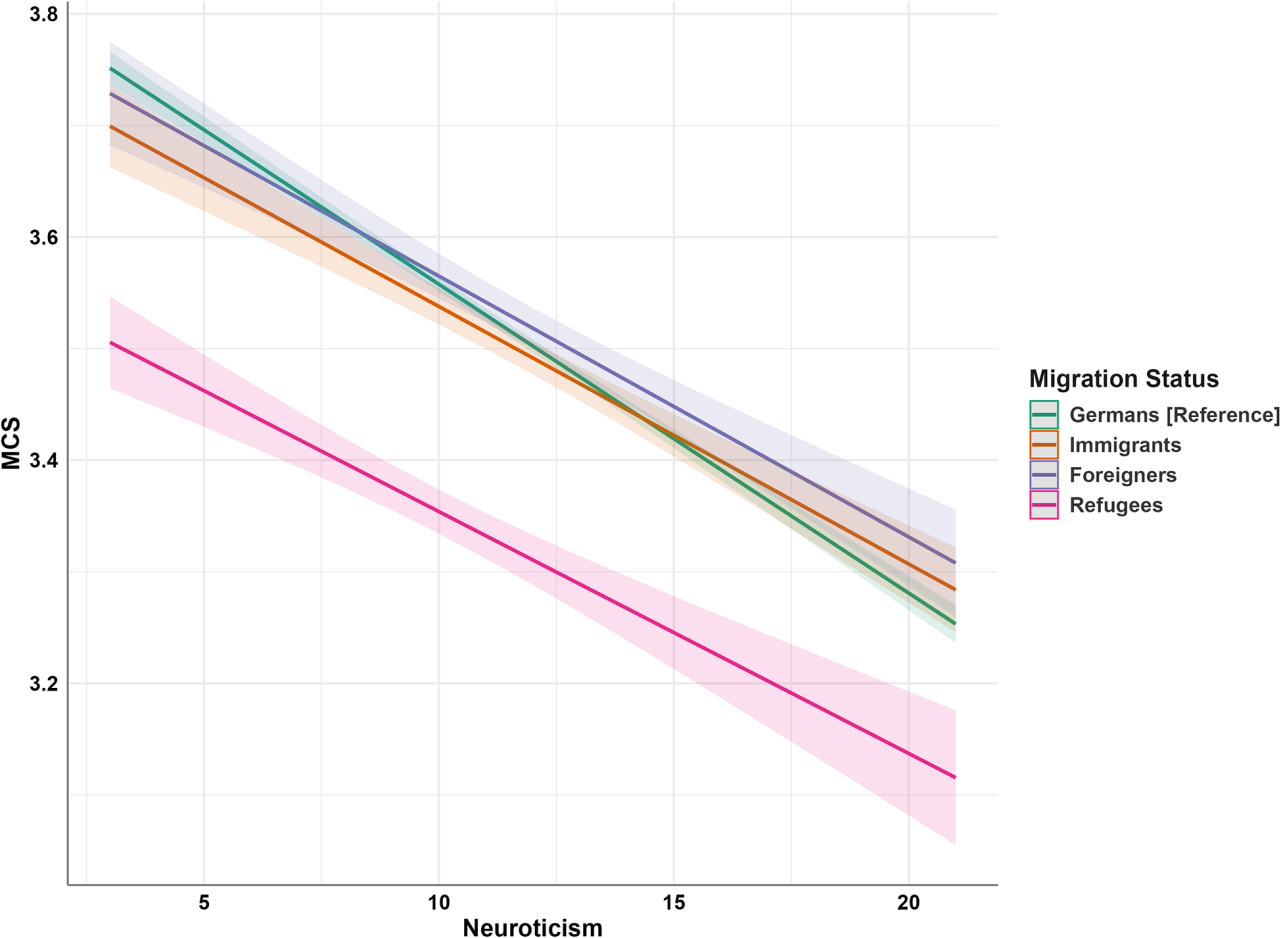
Moderating effect of neuroticism on the relationship between migration status and MCS. Note: MCS = Mental Component Summary of SF-12; reference group = Germans. For significant interactions compare Table 6

Figure 2 illustrates the interaction effect of neuroticism on migration status and MCS. It shows that as neuroticism levels increase, MCS decreases. Germans exhibit a more pronounced decline compared to immigrants and refugees (Hypothesis 3).

**Fig. 3:**
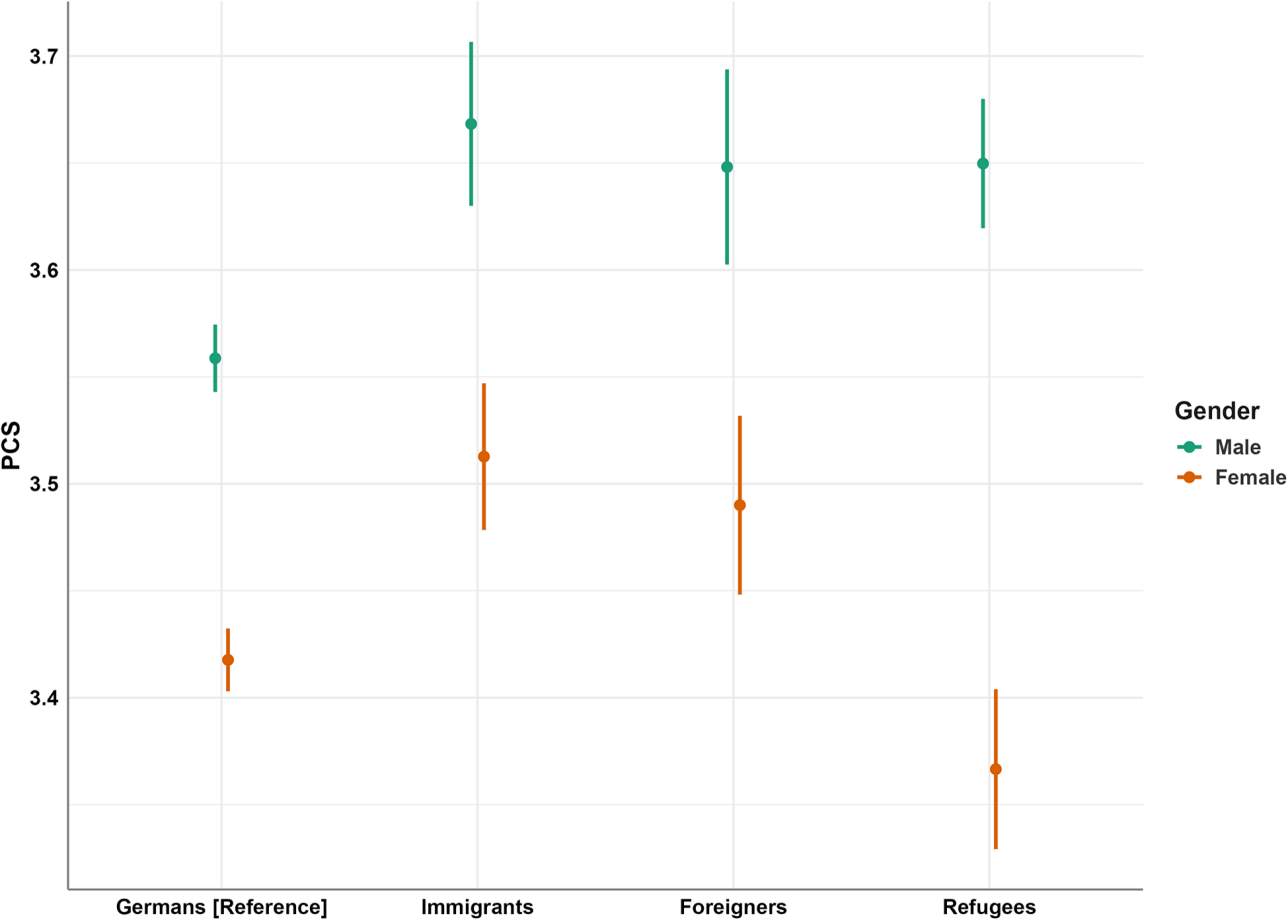
Interaction plot of the moderating effect of gender on the relationship between migration status and PCS. Note: PCS = Physical Component Summary of SF-12; reference group = Germans

In Model 3 (Table 6), the significant association of migration status on SF-12, MCS, and PCS remain unchanged compared to Model 1 (refer to Table 4). Females report significantly lower scores on the SF-12 (β= − 0.22, *p* <.001), MCS (β = − 0.19, *p* <.001), and PCS (β = − 0.18, *p* <.001) compared to males (Hypothesis 4). Female refugees, in particular, show significantly lower SF-12 scores compared to German females (β = − 0.09, *p* =.013). and significant effects are also observed for MCS (β = 0.12, *p* <.001) and PCS (β= − 0.18, *p* <.001). The effect sizes of the regression models increased slightly with higher values for MCS, yet overall they remain low ranging from *R²-*adj = 0.014 for PCS to *R²*-adj = 0.025 for MCS (Table 6). Further details from a post-hoc Tukey test are available in the supplement (Supplement Table S8). Figure 3 illustrates the association of gender, migrations status, and PCS (refer to Table 6) and highlights a significant interaction effect for female refugees compared to male refugees on PCS (Hypothesis 4).

**Table 6:** Regression analyses of SF-12, MCS, and PCS on migration status with gender as moderator

	SF 12		MCS		PCS	
*Predictors*	β (*CI*)	*P*	β (*CI*)	*p*	β (*CI*)	*p*
Intercept	0.12 (0.10 – 0.14)	**< 0.001**	0.16 (0.14 – 0.19)	**< 0.001**	0.06 (0.04 – 0.09)	**< 0.001**
Immigrants	0.09 (0.04 – 0.14)	**0.001**	− 0.05 (-0.11 – 0.00)	0.054	0.14 (0.09 – 0.20)	**< 0.001**
Foreigners	0.08 (0.01 – 0.14)	**0.018**	− 0.02 (-0.08 – 0.04)	0.474	0.12 (0.05 – 0.18)	**< 0.001**
Refugees	− 0.11 (-0.16 – − 0.07)	**< 0.001**	− 0.46 (-0.51 – − 0.42)	**< 0.001**	0.12 (0.07 – 0.16)	**< 0.001**
Gender [female]	− 0.22 (-0.25 – − 0.19)	**< 0.001**	− 0.19 (-0.22 – − 0.16)	**< 0.001**	− 0.18 (-0.21 – − 0.16)	**< 0.001**
Immigrants x gender [female]	− 0.02 (-0.10 – 0.05)	0.538	− 0.01 (-0.08 – 0.06)	0.733	− 0.02 (-0.09 – 0.05)	0.609
Foreigners x gender [female]	− 0.01 (-0.09 – 0.08)	0.852	0.02 (-0.07 – 0.10)	0.674	− 0.02 (-0.11 – 0.06)	0.609
Refugees x gender [female]	− 0.09 (-0.16 – − 0.02)	**0.013**	0.12 (0.06 – 0.19)	**< 0.001**	− 0.18 (-0.25 – − 0.12)	**< 0.001**
Observations	29,196		29,411		29,364	
*R*^*2*^ / *R*^*2*^ adjusted	0.017 / 0.017		0.025 / 0.025		0.014 / 0.014	

Note. SF-12 = Summary Score of SF-12; MCS = Mental Component Summary of SF-12; PCS = Physical Component Summary of SF-12; CI = Standardized CI; reference groups = Germans and men

In Model 4 (Table 7), for SF-12, there is a significant effect for female foreigners (β = − 0.14; *p* =.004) and female refugees (β = − 0.16; *p* =.002) in the interaction with neuroticism compared to their respective reference groups. On the MCS scale, there is a significant negative interaction observed for female foreigners (β = − 0.13; *p* =.006) and female refugees (β = − 0.24; *p* <.001) with neuroticism compared to their reference groups. Similarly, for the PCS scale, the interaction between female foreigners and neuroticism is also significantly negatively associated (β = − 0.10; *p* =.028; Table 7, Hypothesis 5).

**Table 7:** Regression analyses of SF-12, MCS, and PCS on migration status with neuroticism and gender as moderators

	SF 12		MCS			PCS	
*Predictors*	β (*CI*)	*p*		β (*CI*)	*p*	β (*CI*)	*p*
Intercept	0.04 (0.02 – 0.06)	**< 0.001**		0.08 (0.06 – 0.10)	**< 0.001**	0.01 (-0.01 – 0.03)	0.347
Immigrants	0.14 (0.08 – 0.19)	**< 0.001**		− 0.02 (-0.08 – 0.04)	0.515	0.18 (0.12 – 0.24)	**< 0.001**
Foreigners	0.15 (0.09 – 0.22)	**< 0.001**		0.06 (-0.01 – 0.13)	0.090	0.17 (0.10 – 0.24)	**< 0.001**
Refugees	− 0.18 (-0.25 – − 0.11)	**< 0.001**		− 0.49 (-0.56 – − 0.42)	**< 0.001**	0.04 (-0.03 – 0.11)	0.238
Neuroticism	− 0.30 (-0.33 – − 0.28)	**< 0.001**		− 0.26 (-0.28 – − 0.24)	**< 0.001**	− 0.26 (-0.28 – − 0.24)	**< 0.001**
Gender [female]	− 0.11 (-0.14 – − 0.08)	**< 0.001**		− 0.10 (-0.13 – − 0.07)	**< 0.001**	− 0.09 (-0.12 – − 0.06)	**< 0.001**
Immigrants x Neuroticism	0.00 (-0.06 – 0.06)	0.941		0.01 (-0.04 – 0.07)	0.636	− 0.01 (-0.07 – 0.05)	0.804
Foreigners x Neuroticism	0.10 (0.03 – 0.17)	**0.007**		0.11 (0.04 – 0.19)	**0.002**	0.07 (-0.00 – 0.14)	0.064
Refugees x Neuroticism	0.09 (0.03 – 0.15)	**0.004**		0.14 (0.08 – 0.20)	**< 0.001**	0.05 (-0.02 – 0.11)	0.145
Immigrants x Gender [female]	− 0.03 (-0.11 – 0.05)	0.441		− 0.03 (-0.11 – 0.04)	0.411	− 0.02 (-0.10 – 0.06)	0.644
Foreigners x Gender [female]	− 0.02 (-0.12 – 0.07)	0.631		− 0.03 (-0.12 – 0.07)	0.579	− 0.02 (-0.11 – 0.08)	0.711
Refugees x Gender [female]	− 0.09 (-0.20 – 0.01)	0.087		0.05 (-0.05 – 0.16)	0.332	− 0.15 (-0.26 – − 0.05)	**0.005**
Neuroticism x Gender [female]	0.04 (0.01 – 0.07)	**0.011**		0.04 (0.01 – 0.07)	**0.011**	0.03 (0.00 – 0.06)	**0.046**
Immigrants x Neuroticism x Gender [female]	0.03 (-0.04 – 0.11)	0.383		0.05 (-0.03 – 0.12)	0.219	0.02 (-0.05 – 0.10)	0.533
Foreigners x Neuroticism x Gender [female]	− 0.14 (-0.23 – − 0.04)	**0.004**		− 0.13 (-0.22 – − 0.04)	**0.006**	− 0.10 (-0.20 – − 0.01)	**0.028**
Refugees x Neuroticism x Gender [female]	− 0.16 (-0.26 – − 0.06)	**0.002**		− 0.24 (-0.34 – − 0.14)	**< 0.001**	− 0.07 (-0.17 – 0.03)	0.177
Observations	24,032		24,189			24,137	
*R*^*2*^ / *R*^*2*^ adjusted	0.089 / 0.088		0.069 / 0.068			0.067 / 0.066	

Note. SF-12 = Summary Score of SF-12; MCS = Mental Component Summary of SF-12; PCS = Physical Component Summary of SF-12; CI = Standardized CI; reference groups = Germans and men

**Fig. 4:**
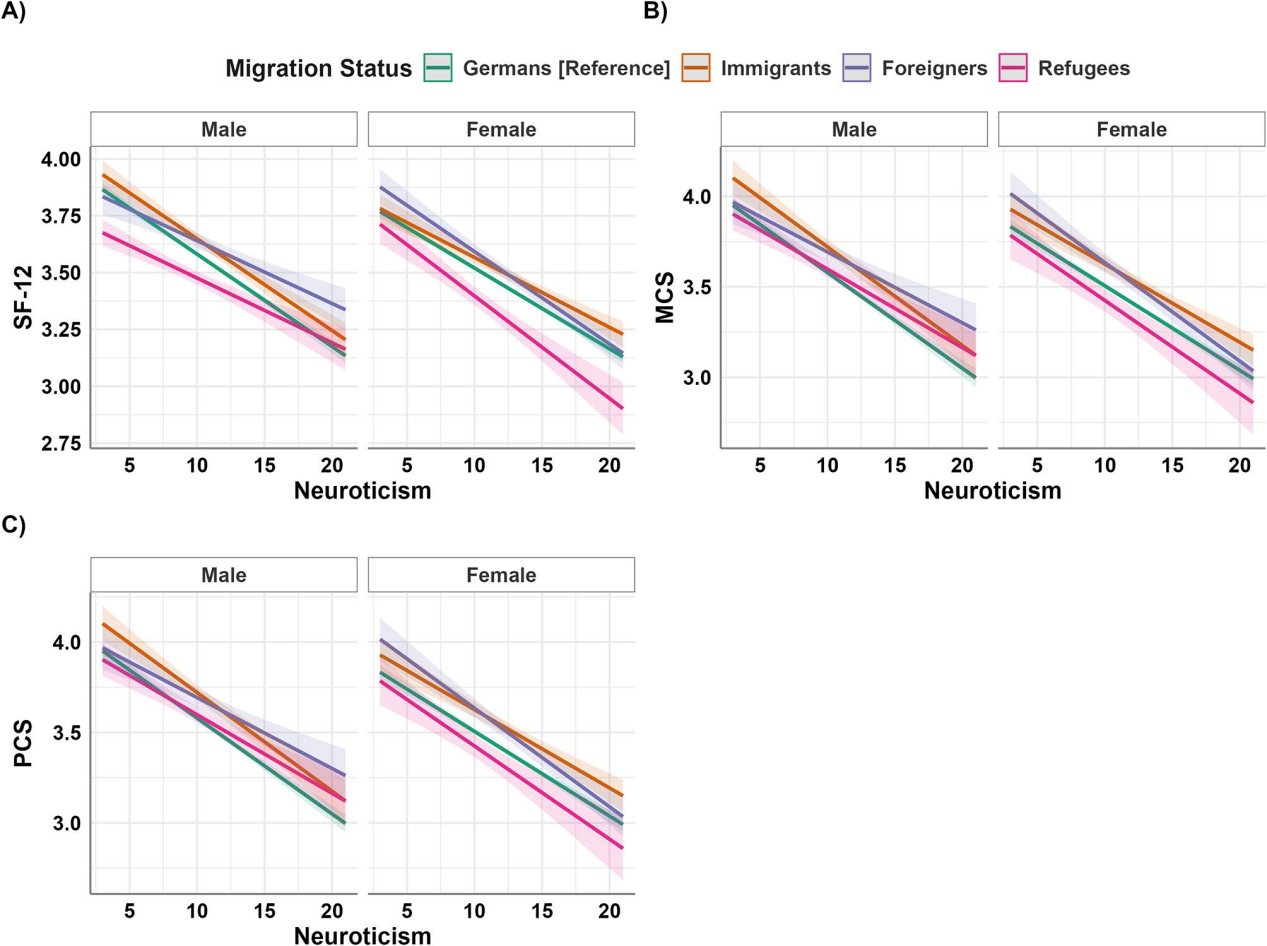
Moderating effect of neuroticism on the relationship between migration status and SF-12,* MCS*, and PCS stratified by gender. Note: SF-12 panel **A**); MCS panel **B**); PCS panel **C**). reference groups = Germans and men. The panels are stratified for gender, allowing the interaction effects to be more clearly illustrated. For the significant interactions, please refer to Table 7

Figure 4 illustrates these interaction effects. Figure 4A shows that female refugees and foreigners experience significant SF-12 declines with increasing neuroticism, contrasting with male refugees and female Germans. Figure 4B reveals distinct patterns between female refugees and foreigners compared to males and Germans in MCS. Specifically, in Fig. 4B, male refugees and foreigners maintain MCS levels despite neuroticism increases, whereas male immigrants and Germans are more negatively affected. Particularly, female refugees with high neuroticism levels experience low MCS scores. Figure 4C highlights the heightened impact of neuroticism on female foreigners and PCS (Hypothesis 5).

## Discussion

In our study exploring the moderating role of neuroticism on the relationship between migration status and HRQoL using SOEP data, we categorized participants into four groups: Germans, immigrants, foreigners, and refugees. Our findings show that refugees reported the lowest HRQoL compared to all groups, particularly in mental health. Female refugees consistently displayed the poorest HRQoL across both mental and physical health domains.

Crucially, the measurement invariance analysis confirmed scalar invariance of the SF-12 across Germans, immigrants, foreigners, and refugees. Because both the factor loadings and item intercepts were equivalent, we can be confident that the observed mean differences in HRQoL reflect true latent differences rather than measurement artefacts or cultural response biases. Establishing scalar invariance is a methodological prerequisite for meaningful group comparisons; it safeguards our interpretations and underpins the robustness of the gender- and migration-related disparities discussed below.

Regarding mental HRQoL, Germans differed significantly from foreigners but not from immigrants, while immigrants do not differ significantly from foreigners. This suggests that immigrants and foreigners may have similar levels of mental HRQoL, possibly due to the higher social integration levels observed among these groups, enhanced by e.g., higher employment rates or longer stay of duration in the host country fostering social interactions compared to refugees.

In terms of the physical component of HRQoL, Germans reported significantly lower physical HRQoL compared to immigrants, foreigners, and refugees. However, there were no significant differences among immigrants, foreigners, and refugees in PCS scores. Our findings align with previous research using GSOEP data, particularly regarding the physical health outcomes among male refugees. For instance, studies by Tibubos and Kröger have similarly identified higher physical health scores among male refugees, potentially attributed to their younger average age [16]. Importantly, our study expands on this by including refugees beyond 2013 and providing a more detailed categorization of migration statuses, distinguishing between immigrants and foreigners living in Germany. Foreigners in our study encompass individuals from EU countries and those with permanent residence permits, among others.

In line with gender-specific findings on neuroticism, across all migration groups women reported significantly higher levels of neuroticism compared to men. Among all migration groups, refugees showed the lowest neuroticism levels. Most refugees in our sample come from Middle Eastern or African countries (e.g., Syria, Afghanistan, Iraq, Eritrea). Cross-cultural comparisons of neuroticism [44] indicate the lowest scores in samples from Africa, while people from Middle East have similar neuroticism levels to those in Western Europe, including Germany. Therefore, differences in mean neuroticism levels cannot be attributed to cross-cultural or regional differences alone (for further discussion on cross-cultural differences in neuroticism, see [45]. To our knowledge, our study is the first to systematically evaluate neuroticism (and other dimension of the OCEAN model) across different migration groups, including refugees. Future research should explore whether low neuroticism increases the likelihood of escaping from war conflict or persecution, overcoming the challenges of migration, and adapting to new environments in the sense of elevated resilience.

We found negative correlations between neuroticism and HRQoL, as well as its subscales, across all groups. This consistent negative relationship indicates that higher neuroticism levels are associated with lower HRQoL, affecting both its mental and physical components. For the relationship of mental HRQoL and migration status, neuroticism showed a moderating influence only among refugees and immigrants compared to Germans, suggesting a buffering effect of neuroticism among immigrants and refugees.

The finding that neuroticism has a distinct impact on all aspects of HRQoL for female refugees underscores the need to consider gender-specific challenges in this population. Female refugees often encounter additional barriers compared to their male counterparts, such as gender-based violence, greater caregiving responsibilities, and cultural barriers to accessing healthcare [5]. High neuroticism may amplify the impact of these barriers, further reducing their QoL. For female refugees already dealing with significant stressors related to displacement, adaptation to a new environment, and potential trauma from pre-migration experiences, high neuroticism could exacerbate these challenges, leading to poorer health outcomes. The convergence of mental HRQoL between female immigrants and female Germans as neuroticism increases suggests that neuroticism may act as a leveling factor, reducing the disparities in HRQoL between these groups. This implies that the coping mechanisms and support systems available to female Germans are similarly accessed or effective for female immigrants, particularly for those with high neuroticism.

### Conclusion and outlook

Successful immigration and acculturation are complex processes depending on multiple factors such as migration motives, gender, age, socialization, and learning [46, 47]. The complexity of these processes makes comprehensive assessment challenging. Moreover, understanding concepts related to migration-sensitive health monitoring is crucial [46, 47]. Importantly, this study included a diverse sample representing various migration statuses and demographics. This diversity allows for a comprehensive exploration of a wide range of experiences related to immigration and acculturation.

Longitudinal studies – particularly transition research that traces individual migration trajectories from asylum seeker, through recognised refugee status, to more permanent residency, thereby pinpointing the timing of HRQoL shifts and the stage-specific protective factors (e.g., low neuroticism) that moderate them – are needed to investigate whether refugees, over time, converge in their HRQoL towards foreigners and eventually to immigrants. We anticipate distinct trajectories for the PCS and MCS components. The MCS is likely to improve with increasing stability and resources (e.g., through asylum acceptance, work permits) while the PCS may remain stable due to the Healthy Migrant Effect, particularly affecting men and physical health [48]. Thus, future studies should account for age, gender, and cohort effects.

Overall, this study highlights the moderating role of neuroticism in relation to migration status and HRQoL, utilizing a large cross-sectional sample that distinguishes between Germans, immigrants, foreigners, and refugees. A notable finding is the varied association of neuroticism on HRQoL across these groups. Refugees, particularly those with low neuroticism, exhibit resilience, potentially buffering the adverse effects of migration on HRQoL. Conversely, higher neuroticism is associated with poorer health outcomes, especially among female refugees facing additional gender-specific challenges. Additionally, immigrants show comparable mental HRQoL to Germans, potentially due to greater social integration. However, neuroticism moderates these effects differently depending on people’s migration status. Given the complexity of immigration and acculturation processes, longitudinal studies are needed to explore these dynamics comprehensively over time.

## Electronic supplementary material

Below is the link to the electronic supplementary material.

Supplementary Material 1

## Data Availability

The data used in this study are derived from the Socio-Economic Panel (SOEP), specifically SOEP Core, Version 37, including data from 1984 to 2020 (SOEP-Core v37, EU-Edition, 2022). Access to these data is restricted and can be requested through a cooperation with the SOEP archive at DIW Berlin. Link: https://www.diw.de/en/diw_01.c.601584.en/data_access.html.Please cite the data as follows: Main Data: Socio-Economic Panel (SOEP), Version 37, Data from 1984 to 2020 (SOEP-Core v37, EU-Edition). 2022. DOI: 10.5684/soep.core.v37eu. Additionally: Migration samples were included in our analysis, please also cite: IAB-SOEP Migration Samples (M1, M2), data from 2013 to 2020, DOI: 10.5684/soep.iab-soep-mig.2020.Refugee samples were included, please cite: IAB-BAMF-SOEP Survey of Refugees (M3-M5), data from 2016 to 2020, DOI: 10.5684/soep.iab-bamf-soep-mig.2020.
