# Supplementary material for: The moderating effect of neuroticism on the relationship of migration status and health-related quality of life in Germany: a population-based study
Source: Health Qual Life Outcomes. 2025 Jun 6;23:58. doi: 10.1186/s12955-025-02380-0 (PMC12142877; doi:10.1186/s12955-025-02380-0)
Supplement: Supplementary file 1 — Supplementary Material 1 [file 12955_2025_2380_MOESM1_ESM.docx]

**Supplemental material to the manuscript**

**The Moderating Effect of Neuroticism on the Relationship of Migration Status and Health-Related Quality of Life in Germany: A Population-Based Study**

**Table S1**

*Country of Origins in the migration Groups*

|  | **Germans *n* (%)** | **Immigrants *n* (%)** | **Foreigners *n* (%)** | **Refugees *n* (%)** |
| --- | --- | --- | --- | --- |
| Germany | 19,749 (100.00%) | 1,407 (40.30%) |  |  |
| Turkey |  | 135 (3.87%) | 243 (9.88%) | 3 (0.07%) |
| Former Yugoslavia |  | 6 (0.17%) | 2 (0.08%) |  |
| Greece |  | 10 (0.29%) | 74 (3.01%) |  |
| Italy |  | 22 (0.63%) | 138 (5.61%) |  |
| Spain |  | 7 (0.20%) | 43 (1.75%) |  |
| Austria |  | 17 (0.49%) | 45 (1.83%) | 1 (0.02%) |
| France |  | 15 (0.43%) | 31 (1.26%) |  |
| Denmark |  | 1 (0.03%) | 10 (0.41%) |  |
| Great Britain |  | 5 (0.14%) | 17 (0.69%) |  |
| Sweden |  | 2 (0.06%) | 7 (0.28%) |  |
| Norway |  |  | 2 (0.08%) |  |
| Finland |  |  | 5 (0.20%) |  |
| USA |  | 9 (0.26%) | 21 (0.85%) |  |
| Switzerland |  | 6 (0.17%) | 16 (0.65%) |  |
| Chile |  | 7 (0.20%) | 5 (0.20%) |  |
| Romania |  | 141 (4.04%) | 177 (7.20%) | 2 (0.05%) |
| Poland |  | 316 (9.05%) | 240 (9.76%) |  |
| Korea |  | 1 (0.03%) | 2 (0.08%) |  |
| Iran |  | 31 (0.89%) | 17 (0.69%) | 112 (2.62%) |
| Indonesia |  |  | 2 (0.08%) |  |
| Hungary |  | 6 (0.17%) | 58 (2.36%) |  |
| Bolivia |  | 1 (0.03%) |  |  |
| Portugal |  | 2 (0.06%) | 20 (0.81%) |  |
| Bulgaria |  | 12 (0.34%) | 87 (3.54%) |  |
| Syria |  | 25 (0.72%) | 130 (5.28%) | 2,381 (55.79%) |
| Czech Republic |  | 24 (0.69%) | 16 (0.65%) |  |
| Russia |  | 375 (10.74%) | 117 (4.76%) | 50 (1.17%) |
| Mexico |  | 6 (0.17%) | 5 (0.20%) |  |
| Argentina |  | 5 (0.14%) | 3 (0.12%) |  |
| Benin |  | 1 (0.03%) |  |  |
| Philippines |  | 12 (0.34%) | 4 (0.16%) |  |
| Israel |  | 1 (0.03%) | 1 (0.04%) |  |
| Japan |  |  | 4 (0.16%) |  |
| Australia |  | 3 (0.09%) | 1 (0.04%) |  |
| India |  | 7 (0.20%) | 19 (0.77%) | 4 (0.09%) |
| Afghanistan |  | 16 (0.46%) | 47 (1.91%) | 488 (11.43%) |
| Thailand |  | 3 (0.09%) | 14 (0.57%) |  |
| Jamaica |  | 1 (0.03%) | 3 (0.12%) |  |
| Saudi Arabia |  |  | 2 (0.08%) | 10 (0.23%) |
| Ethiopia |  | 2 (0.06%) | 3 (0.12%) | 22 (0.52%) |
| Colombia |  | 4 (0.11%) | 5 (0.20%) |  |
| Ghana |  | 5 (0.14%) | 10 (0.41%) | 7 (0.16%) |
| Bangladesh |  | 4 (0.11%) | 3 (0.12%) | 4 (0.09%) |
| Venezuela |  | 2 (0.06%) | 1 (0.04%) | 1 (0.02%) |
| Tunisia |  | 9 (0.26%) | 10 (0.41%) | 1 (0.02%) |
| Mauritius |  |  | 1 (0.04%) |  |
| Nigeria |  | 7 (0.20%) | 9 (0.37%) | 34 (0.80%) |
| Canada |  | 1 (0.03%) | 1 (0.04%) |  |
| New Zealand |  |  | 2 (0.08%) |  |
| Cuba |  | 2 (0.06%) | 5 (0.20%) |  |
| Iraq |  | 30 (0.86%) | 67 (2.72%) | 571 (13.38%) |
| Brazil |  | 7 (0.20%) | 12 (0.49%) |  |
| Peru |  | 4 (0.11%) | 4 (0.16%) |  |
| Sri Lanka |  | 11 (0.32%) | 7 (0.28%) | 6 (0.14%) |
| Nepal |  | 1 (0.03%) | 1 (0.04%) |  |
| Morocco |  | 22 (0.63%) | 21 (0.85%) | 6 (0.14%) |
| China |  | 5 (0.14%) | 7 (0.28%) |  |
| Ireland |  | 1 (0.03%) | 4 (0.16%) |  |
| Moldova |  | 2 (0.06%) | 4 (0.16%) | 1 (0.02%) |
| Kazakhstan |  | 386 (11.06%) | 40 (1.63%) |  |
| Albania |  | 7 (0.20%) | 8 (0.33%) | 13 (0.30%) |
| Lebanon |  | 11 (0.32%) | 11 (0.45%) | 27 (0.63%) |
| Kyrgyzstan |  | 28 (0.80%) | 5 (0.20%) |  |
| Ukraine |  | 68 (1.95%) | 63 (2.56%) | 7 (0.16%) |
| Algeria |  | 2 (0.06%) | 2 (0.08%) | 4 (0.09%) |
| Mozambique |  | 1 (0.03%) |  |  |
| Egypt |  | 4 (0.11%) | 2 (0.08%) | 10 (0.23%) |
| Tajikistan |  | 9 (0.26%) | 2 (0.08%) | 3 (0.07%) |
| Vietnam |  | 4 (0.11%) | 6 (0.24%) |  |
| Somalia |  | 1 (0.03%) | 5 (0.20%) | 52 (1.22%) |
| Pakistan |  | 15 (0.43%) | 20 (0.81%) | 64 (1.50%) |
| South Africa |  | 5 (0.14%) | 1 (0.04%) |  |
| United Arab Emirates |  |  | 1 (0.04%) | 8 (0.19%) |
| El Salvador |  | 1 (0.03%) |  |  |
| Eritrea |  | 2 (0.06%) | 4 (0.16%) | 204 (4.78%) |
| Jordan |  | 6 (0.17%) | 1 (0.04%) | 2 (0.05%) |
| Singapore |  | 1 (0.03%) |  |  |
| Burkina Faso |  |  |  | 2 (0.05%) |
| Zambia |  |  | 1 (0.04%) |  |
| Ecuador |  | 1 (0.03%) | 2 (0.08%) |  |
| Uzbekistan |  | 12 (0.34%) | 4 (0.16%) | 2 (0.05%) |
| Stateless |  |  |  | 1 (0.02%) |
| Laos |  | 1 (0.03%) |  |  |
| Estonia |  | 3 (0.09%) |  |  |
| Angola |  |  | 3 (0.12%) | 1 (0.02%) |
| Latvia |  | 1 (0.03%) | 9 (0.37%) |  |
| Malaysia |  |  | 2 (0.08%) |  |
| Namibia |  | 2 (0.06%) |  |  |
| Montenegro |  |  | 4 (0.16%) | 3 (0.07%) |
| Dominican Republic |  | 2 (0.06%) |  |  |
| Nicaragua |  | 2 (0.06%) | 1 (0.04%) |  |
| Kenya |  | 3 (0.09%) | 3 (0.12%) | 1 (0.02%) |
| Libya |  | 1 (0.03%) |  | 19 (0.45%) |
| Botswana |  | 2 (0.06%) |  |  |
| Haiti |  |  | 1 (0.04%) |  |
| Luxembourg |  |  | 2 (0.08%) |  |
| Belgium |  | 5 (0.14%) | 3 (0.12%) |  |
| Netherlands |  | 9 (0.26%) | 36 (1.46%) |  |
| Croatia |  | 8 (0.23%) | 36 (1.46%) |  |
| Bosnia/Herzegovina |  | 14 (0.40%) | 52 (2.11%) | 1 (0.02%) |
| Macedonia |  | 2 (0.06%) | 27 (1.10%) | 8 (0.19%) |
| Slovenia |  | 5 (0.14%) | 8 (0.33%) |  |
| Slovakia |  | 3 (0.09%) | 15 (0.61%) |  |
| Paraguay |  | 1 (0.03%) |  |  |
| Guinea |  |  | 3 (0.12%) | 8 (0.19%) |
| Kuwait |  | 2 (0.06%) | 1 (0.04%) | 11 (0.26%) |
| Ivory Coast |  | 1 (0.03%) |  |  |
| Samoa |  | 1 (0.03%) | 1 (0.04%) |  |
| Azerbaijan |  | 6 (0.17%) | 16 (0.65%) | 9 (0.21%) |
| Belarus |  | 10 (0.29%) | 15 (0.61%) |  |
| Uruguay |  | 1 (0.03%) |  |  |
| Uganda |  |  |  | 1 (0.02%) |
| Mali |  |  | 1 (0.04%) | 2 (0.05%) |
| Cameroon |  | 2 (0.06%) | 4 (0.16%) | 5 (0.12%) |
| Kosovo-Albania |  | 29 (0.83%) | 100 (4.07%) | 18 (0.42%) |
| Georgia |  | 5 (0.14%) | 4 (0.16%) | 12 (0.28%) |
| Sudan |  |  | 1 (0.04%) | 11 (0.26%) |
| Congo |  | 1 (0.03%) | 2 (0.08%) | 2 (0.05%) |
| Togo |  | 2 (0.06%) | 5 (0.20%) |  |
| Mongolia |  |  |  | 2 (0.05%) |
| Lithuania |  | 5 (0.14%) | 12 (0.49%) |  |
| Chad |  |  |  | 2 (0.05%) |
| Armenia |  | 1 (0.03%) | 10 (0.41%) | 10 (0.23%) |
| Yemen |  |  |  | 2 (0.05%) |
| Palestine |  | 8 (0.23%) | 2 (0.08%) | 11 (0.26%) |
| Taiwan |  |  | 3 (0.12%) |  |
| Turkmenistan |  | 3 (0.09%) | 1 (0.04%) |  |
| Sierra Leone |  |  | 1 (0.04%) |  |
| Senegal |  |  | 1 (0.04%) |  |
| Serbia |  | 18 (0.52%) | 84 (3.41%) | 18 (0.42%) |
| Gambia |  | 1 (0.03%) |  | 7 (0.16%) |
| Cambodia |  | 1 (0.03%) |  |  |
| Suriname |  |  | 2 (0.08%) |  |
| Guyana |  |  | 1 (0.04%) |  |
| Zimbabwe |  |  | 1 (0.04%) |  |
| Lesotho |  | 1 (0.03%) |  |  |
| Qatar |  |  | 1 (0.04%) |  |
| Kosovo |  |  | 2 (0.08%) |  |
| NA |  | 8 (0.23%) | 1 (0.04%) | 1 (0.02%) |
| **Total** | **19,749 (100%)** | **3,491 (100%)** | **2,460 (100%)** | **4,268 (100%)** |

**S2 Measurement Invariance**

Further, we aimed to test measurement invariance of the SF-12 across migration groups using recommended procedures (Cheung & Rensvold, 2002; Milfont & Fischer, 2010). Specifically, we examined measurement invariance employing a second-order factor model, comprising two first-order factors representing the Physical Component Summary (PCS) and the Mental Component Summary (MCS), and a second-order factor reflecting HRQoL (SF-12). We compared increasingly constrained models to evaluate progressively stricter levels of invariance stepwise. First, we assessed weak (metric) invariance by constraining factor loadings (first- and second-order) to be equal across migration groups. Second, we tested strong (scalar) invariance by additionally constraining item intercepts to equality. Third, we examined strict invariance by constraining item residual variances to be equal across groups. Following recommendations by Milfont and Fischer (2010), model comparisons were evaluated using the robust χ²-difference test (Satorra-Bentler scaled difference test; Satorra & Bentler, 2001), supplemented by assessing changes in comparative fit index (CFI) and gamma hat (GH; Steiger, 1989). Ideally, the χ²-difference test should not yield significant results, and differences in CFI and GH between the compared models should be ≤ .01 to indicate invariance.

Based on the results presented in Table S2, scalar measurement invariance of the SF-12 across migration groups can be assumed with high confidence. Although the robust χ²-difference tests were significant, the observed changes in supplementary fit indices (ΔCFI ≤ .01; ΔGH ≤ .01) were well within the recommended thresholds (Milfont & Fischer, 2010). Thus, scalar invariance for both SF-12 components (PCS and MCS) was supported, allowing us to perform complex statistical analyses involving these latent health constructs across migration groups.

**Table S2**

*Measurement Invariance across migration groups*

| Model | χ² (*df*) | Δχ² | Δ*df* | *p* | *CFI* | Δ*CFI* | *GH* | Δ*GH* |
| --- | --- | --- | --- | --- | --- | --- | --- | --- |
| Configural invariance | 43,359.95 (208) |  |  | *** | .791 |  | .802 |  |
| Metric invariance | 43,894.68 (241) | 471.71 | 33 | *** | .788 | .003 | .801 | .001 |
| Scalar invariance | 45,630.44 (268) | 1546.3 | 27 | *** | .780 | .008 | .794 | .007 |
| Strict invariance | 48,101.92 (304) | 1,227.0 | 36 | *** | .768 | .012 | .786 | .008 |

**Note.** Δχ² = Chi-square difference based on the robust χ²-difference test (Satorra-Bentler scaled difference test; Satorra & Bentler, 2001).

**Table S3**

*R-Packages used for the Analysis*

| Package Name | Version | Reference |
| --- | --- | --- |
| janitor | 2.2.0 | Firke S (2023). _janitor: Simple Tools for Examining and Cleaning Dirty Data_. R package version 2.2.0, https://CRAN.R-project.org/package=janitor. |
| labelled | 2.13.0 | Larmarange J (2024). _labelled: Manipulating Labelled Data_. R package version 2.13.0, https://CRAN.R-project.org/package=labelled. |
| psych | 2.4.3 | William Revelle (2024). _psych: Procedures for Psychological, Psychometric, and Personality Research_. Northwestern University, Evanston, Illinois. R package version 2.4.3, https://CRAN.R-project.org/package=psych. |
| patchwork | 1.2.0 | Pedersen T (2024). _patchwork: The Composer of Plots_. R package version 1.2.0,<https://CRAN.R-project.org/package=patchwork. |
| sjstats | 0.19.0 | Lüdecke D (2024). _sjstats: Statistical Functions for Regression Models (Version 0.19.0)_. doi:10.5281/zenodo.1284472. |
| sjmisc | 2.8.10 | Lüdecke D (2018). “sjmisc: Data and Variable Transformation Functions.” _Journal of Open Source Software_, *3*(26), 754. doi:10.21105/joss.00754. |
| sjPlot | 2.8.16 | Lüdecke D (2024). _sjPlot: Data Visualization for Statistics in Social Science_. R package version 2.8.16, https://CRAN.R-project.org/package=sjPlot. |
| dplyr | 1.1.4 | Wickham H, François R, Henry L, Müller K, Vaughan D (2023). _dplyr: A Grammar of Data Manipulation_. R package version 1.1.4, https://CRAN.R-project.org/package=dplyr. |
| ggplot2 | 3.5.1 | Wickham, H. (2016). ggplot2: Elegant Graphics for Data Analysis. Springer-Verlag New York, 2016. |
| readr | 2.1.5 | Wickham H, Hester J, Bryan J (2024). _readr: Read Rectangular Text Data_. R package version 2.1.5, https://CRAN.R-project.org/package=readr. |
| tidyr | 1.3.1 | Wickham H, Vaughan D, Girlich M (2024). _tidyr: Tidy Messy Data_. R package version 1.3.1, https://CRAN.R-project.org/package=tidyr. |
| haven | 2.5.4 | Wickham H, Miller E, Smith D (2023). _haven: Import and Export 'SPSS', 'Stata' and 'SAS' Files_. R package version 2.5.4, https://CRAN.R-project.org/package=haven. |

**Table S4**

*Correlation matrix of gender, age, and the measures SF-12, its subscales MCS and PCS, and the Big Five domains including neuroticism for Germans*

|  | **Age** | **SF-12** | **MCS** | **PCS** | **O** | **C** | **E** | **A** | **N** |
| --- | --- | --- | --- | --- | --- | --- | --- | --- | --- |
| **Gender** | -.016^*^ | -.116^***^ | -.104^***^ | -.093^***^ | .050^***^ | .124^***^ | .114^***^ | .148^***^ | **.209^***^** |
| **Age** |  | **-.216^***^** | .116^***^ | **-.335^***^** | -.004 | .149^***^ | -.058^***^ | .058^***^ | -.051^***^ |
| **SF-12** |  |  | **.669^***^** | **.922^***^** | .025^**^ | .047^***^ | .062^***^ | .055^***^ | **-.298^***^** |
| **MCS** |  |  |  | **.329^***^** | -.035^***^ | .061^***^ | .021^**^ | .066^***^ | **-.257^***^** |
| **PCS** |  |  |  |  | .050^***^ | .028^***^ | .068^***^ | .035^***^ | **-.245^***^** |
| **O** |  |  |  |  |  | **.179^***^** | **.320^***^** | .127^***^ | -.087^***^ |
| **C** |  |  |  |  |  |  | **.202^***^** | **.245^***^** | -.104^***^ |
| **E** |  |  |  |  |  |  |  | .053^***^ | **-.158^***^** |
| **A** |  |  |  |  |  |  |  |  | -.126^***^ |

*Note.* SF-12 = Summary Score of SF-12; MCS = Mental Component Summary of SF-12; PCS = Physical Component Summary of SF-12;
BFI-S domains: O = Openness; C = Conscientiousness; E = Extraversion; A = Agreeableness; N = Neuroticism; gender was coded 1 = male, 2 = female; computed correlation used pearson-method with listwise-deletion; correlation coefficients > ± .150 are in bold.
*** = *p* < .001; ** = *p* < .01; * = *p* < .05

**Table S5**

*Correlation matrix of gender, age, and the measures SF-12, its subscales MCS and PCS, and the Big Five domains including neuroticism for Immigrants*

|  | **Age** | **SF-12** | **MCS** | **PCS** | **O** | **C** | **E** | **A** | **N** |
| --- | --- | --- | --- | --- | --- | --- | --- | --- | --- |
| **Gender** | .006 | -.121^***^ | -.103^***^ | -.104^***^ | .070^***^ | .105^***^ | .095^***^ | .125^***^ | **.200^***^** |
| **Age** |  | **-.207^***^** | .054^**^ | **-.311^***^** | -.096^***^ | **.193^***^** | -.116^***^ | .070^***^ | -.019 |
| **SF-12** |  |  | **.745^***^** | **.924^***^** | .019 | .064^***^ | .055^**^ | .082^***^ | **-.267^***^** |
| **MCS** |  |  |  | **.433^***^** | -.080^***^ | .090^***^ | .004 | .084^***^ | **-.204^***^** |
| **PCS** |  |  |  |  | .071^***^ | .035 | .072^***^ | .062^***^ | **-.244^***^** |
| **O** |  |  |  |  |  | .143^***^ | **.351^***^** | .117^***^ | -.059^**^ |
| **C** |  |  |  |  |  |  | **.213^***^** | **.300^***^** | -.122^***^ |
| **E** |  |  |  |  |  |  |  | .094^***^ | **-.162^***^** |
| **A** |  |  |  |  |  |  |  |  | **-.150^***^** |

**Note**: SF-12 = Summary Score of SF-12; MCS = Mental Component Summary of SF-12; PCS = Physical Component Summary of SF-12;
BFI-S domains: O = Openness; C = Conscientiousness; E = Extraversion; A = Agreeableness; N = Neuroticism; gender was coded 1 = male, 2 = female; computed correlation used pearson-method with listwise-deletion; correlation coefficients > ± .150 are in bold.
*** = *p* < .001; ** = *p* < .01; * = *p* < .05

**Table S6**

*Correlation matrix of gender, age, and the measures SF-12, its subscales MCS and PCS, and the Big Five domains including neuroticism for Foreigner*

|  | **Age** | **SF-12** | **MCS** | **PCS** | **O** | **C** | **E** | **A** | **N** |
| --- | --- | --- | --- | --- | --- | --- | --- | --- | --- |
| **Gender** | -.082^***^ | -.117^***^ | -.104^***^ | -.100^***^ | .062^**^ | .042 | .037 | .108^***^ | **.187^***^** |
| **Age** |  | **-.225^***^** | .001 | -.304^***^ | -.116^***^ | .013 | -.108^***^ | .008 | -.051^*^ |
| **SF-12** |  |  | **.753^***^** | **.932^***^** | .092^***^ | .059^*^ | .089^***^ | .011 | **-.257^***^** |
| **MCS** |  |  |  | **.462^***^** | -.046^*^ | .021 | .012 | .022 | **-.204^***^** |
| **PCS** |  |  |  |  | **.150^***^** | .068^**^ | .114^***^ | .003 | **-.234^***^** |
| **O** |  |  |  |  |  | **.244^***^** | **.308^***^** | .132^***^ | -.101^***^ |
| **C** |  |  |  |  |  |  | **.249^***^** | **.291^***^** | -.138^***^ |
| **E** |  |  |  |  |  |  |  | .043 | -.112^***^ |
| **A** |  |  |  |  |  |  |  |  | -.128^***^ |

*Note.* SF-12 = Summary Score of SF-12; MCS = Mental Component Summary of SF-12; PCS = Physical Component Summary of SF-12;
BFI-S domains: O = Openness; C = Conscientiousness; E = Extraversion; A = Agreeableness; N = Neuroticism; gender was coded 1 = male, 2 = female; computed correlation used pearson-method with listwise-deletion; correlation coefficients > ± .150 are in bold.
*** = *p* < .001; ** = *p* < .01; * = *p* < .05

**Table S7**

*Correlation matrix of gender, age, and the measures SF-12, its subscales MCS and PCS, and the Big Five domains including neuroticism for Refugees*

|  | **Age** | **SF-12** | **MCS** | **PCS** | **O** | **C** | **E** | **A** | **N** |
| --- | --- | --- | --- | --- | --- | --- | --- | --- | --- |
| **Gender** | .001 | -.080^**^ | .002 | -.123^***^ | -.082^**^ | .060^*^ | -.038 | .043 | .099^***^ |
| **Age** |  | **-.192^***^** | -.048 | **-.257^***^** | .028 | .116^***^ | .085^**^ | .144^***^ | .020 |
| **SF-12** |  |  | **.831^***^** | **.917^***^** | .009 | .106^***^ | .083^**^ | .047 | **-.228^***^** |
| **MCS** |  |  |  | **.541^***^** | -.038 | .088^***^ | .029 | .050 | **-.137^***^** |
| **PCS** |  |  |  |  | .041 | .098^***^ | .104^***^ | .036 | **-.246^***^** |
| **O** |  |  |  |  |  | **.291^***^** | **.257^***^** | **.178^***^** | -.095^***^ |
| **C** |  |  |  |  |  |  | **.269^***^** | **.436^***^** | **-.182^***^** |
| **E** |  |  |  |  |  |  |  | **.175^***^** | **-.180^***^** |
| **A** |  |  |  |  |  |  |  |  | **-.179^***^** |

*Note.* SF-12 = Summary Score of SF-12; MCS = Mental Component Summary of SF-12; PCS = Physical Component Summary of SF-12;
BFI-S domains: O = Openness; C = Conscientiousness; E = Extraversion; A = Agreeableness; N = Neuroticism; gender was coded 1 = male, 2 = female; computed correlation used pearson-method with listwise-deletion; correlation coefficients > ± .150 are in bold.
*** = *p* < .001; ** = *p* < .01; * = *p* < .05

**Table S8**

*Post-Hoc-Tukey-Test for Model 2*

| *Group 1* | *Group 2* | *estimate* | *CI* | *p.adj* |  |
| --- | --- | --- | --- | --- | --- |
| Germans | Immigrants | -.027 | -.047 – -.007 | .003 | ** |
| Germans | Foreigners | -.006 | -.030 – .018 | .908 | ns |
| Germans | Refugees | -.167 | -.186 – -.148 | .000 | **** |
| Immigrants | Foreigners | .021 | -.008 – .050 | .256 | ns |
| Immigrants | Refugees | -.140 | -.166 – -.115 | .000 | **** |
| Foreigners | Refugees | -.161 | -.190 – -.133 | .000 | **** |
| Male | Female | -.075 | -.085 – -.065 | .000 | **** |
| Germans:Male | Immigrants:Male | -.023 | -.058 – .013 | .535 | ns |
| Germans:Male | Foreigners:Male | -.010 | -.052 – .032 | .997 | ns |
| Germans:Male | Refugees:Male | -.200 | -.230 – -.171 | .000 | **** |
| Germans:Male | Germans:Female | -.083 | -.102 – -.065 | .000 | **** |
| Germans:Male | Immigrants:Female | -.111 | -.144 – -.079 | .000 | **** |
| Germans:Male | Foreigners:Female | -.085 | -.124 – -.047 | .000 | **** |
| Germans:Male | Refugees:Female | -.230 | -.265 – -.195 | .000 | **** |
| Immigrants:Male | Foreigners:Male | .013 | -.039 – .064 | .995 | ns |
| Immigrants:Male | Refugees:Male | -.178 | -.220 – -.136 | .000 | **** |
| Immigrants:Male | Germans:Female | -.061 | -.096 – -.025 | .000 | **** |
| Immigrants:Male | Immigrants:Female | -.089 | -.133 – -.044 | .000 | **** |
| Immigrants:Male | Foreigners:Female | -.063 | -.111 – -.014 | .003 | ** |
| Immigrants:Male | Refugees:Female | -.207 | -.253 – -.161 | .000 | **** |
| Foreigners:Male | Refugees:Male | -.191 | -.238 – -.143 | .000 | **** |
| Foreigners:Male | Germans:Female | -.073 | -.115 – -.032 | .000 | **** |
| Foreigners:Male | Immigrants:Female | -.101 | -.151 – -.052 | .000 | **** |
| Foreigners:Male | Foreigners:Female | -.075 | -.129 – -.022 | .001 | *** |
| Foreigners:Male | Refugees:Female | -.220 | -.271 – -.169 | .000 | **** |
| Refugees:Male | Germans:Female | .117 | .088 – .146 | .000 | **** |
| Refugees:Male | Immigrants:Female | .089 | .050 – .129 | .000 | **** |
| Refugees:Male | Foreigners:Female | .115 | .071 – .160 | .000 | **** |
| Refugees:Male | Refugees:Female | -.029 | -.071 – .012 | .399 | ns |
| Germans:Female | Immigrants:Female | -.028 | -.060 – .004 | .141 | ns |
| Germans:Female | Foreigners:Female | -.002 | -.040 – .036 | 1.000 | ns |
| Germans:Female | Refugees:Female | -.146 | -.181 – -.112 | .000 | **** |
| Immigrants:Female | Foreigners:Female | .026 | -.021 – .073 | .691 | ns |
| Immigrants:Female | Refugees:Female | -.118 | -.162 – -.074 | .000 | **** |
| Foreigners:Female | Refugees:Female | -.144 | -.193 – -.096 | .000 | **** |

*Note.* *** = *p* < .001; ** = *p* < .01; * = *p* < .05
